# X-linked hypomyelination with spondylometaphyseal dysplasia (H-SMD) associated with mutations in *AIFM1*

**DOI:** 10.1007/s10048-017-0520-x

**Published:** 2017-08-26

**Authors:** Noriko Miyake, Nicole I. Wolf, Ferdy K. Cayami, Joanna Crawford, Annette Bley, Dorothy Bulas, Alex Conant, Stephen J. Bent, Karen W. Gripp, Andreas Hahn, Sean Humphray, Shihoko Kimura-Ohba, Zoya Kingsbury, Bryan R. Lajoie, Dennis Lal, Dimitra Micha, Amy Pizzino, Richard J. Sinke, Deborah Sival, Irene Stolte-Dijkstra, Andrea Superti-Furga, Nicole Ulrick, Ryan J. Taft, Tsutomu Ogata, Keiichi Ozono, Naomichi Matsumoto, Bernd A. Neubauer, Cas Simons, Adeline Vanderver

**Affiliations:** 1 Department of Human Genetics, Yokohama City University Graduate School of Medicine, Fukuura, Kanazawa-ku, Yokohama, 236-0004 Japan; 2 Department of Child Neurology, and Amsterdam Neuroscience, VU University Medical Center, De Boelelaan 1117, 1081 HV Amsterdam, the Netherlands; 3 Department of Clinical Genetics, VU University Medical Center, De Boelelaan 1117, 1081 HV Amsterdam, the Netherlands; 4 Center for Biomedical Research, Faculty of Medicine, Diponegoro University, Semarang, Indonesia; 5 Institute for Molecular Bioscience, The University of Queensland, Brisbane, Australia; 6 University Children’s Hospital, University Medical Center Hamburg Eppendorf, Martinistr. 52, 20246 Hamburg, Germany; 7 Department of Diagnostic Imaging and Radiology, Children’s National Medical Center, Washington, DC USA; 8 Department of Neurology, Children’s National Medical Center, Suite 4800, Washington, DC USA; 9 Division of Medical Genetics, A.I. duPont Hospital for Children/Nemours, Wilmington, DE USA; 10 Department of Pediatric Neurology, Univ.-Klinikum Giessen/Marburg; Standort Giessen, Feulgenstr. 12, 35389 Giessen, Germany; 11 Chesterford Research Park, Illumina, Inc., Little Chesterford, CB10 1XL UK; 12 Department of Pediatrics, Osaka University Graduate School of Medicine, Osaka, Japan; 13 Illumina, Inc, San Diego, CA USA; 14 Psychiatric and Neurodevelopmental Genetics Unit, Massachusetts General Hospital and Harvard Medical School, Boston, MA USA; 15 Stanley Center for Psychiatric Research, Broad Institute, Cambridge, USA; 16 Department of Genetics, University Medical Center Groningen, University of Groningen, Groningen, The Netherlands; 17 Department of Child Neurology, University Hospital Groningen, Groningen, Netherlands; 18 Division of Genetic Medicine, Centre Hospitalier Universitaire Vaudois (CHUV), University of Lausanne, Lausanne, Switzerland; 19 George Washington University School of Medicine, Washington, DC USA; 20 Department of Pediatrics, Hamamatsu University School of Medicine, Hamamatsu, 431-3192 Japan; 21 Children’s Hospital of Philadelphia, Philadelphia, PA USA

**Keywords:** Hypomyelination, Spondylometaphyseal dysplasia, Whole exome sequencing (WES), AIFM1 gene, Mitochondrial leukodystrophy, Myelin

## Abstract

An X-linked condition characterized by the combination of hypomyelinating leukodystrophy and spondylometaphyseal dysplasia (H-SMD) has been observed in only four families, with linkage to Xq25-27, and recent genetic characterization in two families with a common *AIFM1* mutation. In our study, 12 patients (6 families) with H-SMD were identified and underwent comprehensive assessment accompanied by whole-exome sequencing (WES). Pedigree analysis in all families was consistent with X-linked recessive inheritance. Presentation typically occurred between 12 and 36 months. In addition to the two disease-defining features of spondylometaphyseal dysplasia and hypomyelination on MRI, common clinical signs and symptoms included motor deterioration, spasticity, tremor, ataxia, dysarthria, cognitive defects, pulmonary hypertension, nystagmus, and vision loss due to retinopathy. The course of the disease was slowly progressive. All patients had maternally inherited or de novo mutations in or near exon 7 of *AIFM1*, within a region of 70 bp, including synonymous and intronic changes. *AIFM1* mutations have previously been associated with neurologic presentations as varied as intellectual disability, hearing loss, neuropathy, and striatal necrosis, while *AIFM1* mutations in this small region present with a distinct phenotype implicating bone. Analysis of cell lines derived from four patients identified significant reductions in *AIFM1* mRNA and protein levels in osteoblasts. We hypothesize that AIFM1 functions in bone metabolism and myelination and is responsible for the unique phenotype in this condition.

**Electronic supplementary material:**

The online version of this article (doi:10.1007/s10048-017-0520-x) contains supplementary material, which is available to authorized users.

## Introduction

Hypomyelinating leukodystrophies are a heterogeneous group of genetic disorders characterized by a permanent, significant lack of myelin on brain MR imaging [1, 2]. The archetype of a hypomyelinating disorder, Pelizaeus-Merzbacher disease (PMD), is caused by alterations of *PLP1*, encoding the most abundant structural myelin protein, proteolipid protein 1. However, as new genetic techniques have identified novel genetic causes of hypomyelination, genes coding for structural proteins are not commonly identified. Rather, most genes identified encode proteins involved in RNA metabolism and protein synthesis [3]. How mutations in these genes affect central nervous system (CNS), myelination is still not well understood.

A distinctive hypomyelinating leukodystrophy characterized by the combination of hypomyelination and spondylometaphyseal dysplasia (H-SMD) was first described in one family in 2006 [4], then in an additional patient in 2013 (MIM 300232) [5]. Myelin deficit was confirmed in the brain tissue of a deceased patient, with reduced numbers of oligodendrocytes accompanied by astrocytosis [4]. Although an X-linked pattern of inheritance and linkage to Xq25-27 had already been identified in the first family,^4, 6^ H-SMD could not be associated to a specific causative gene until recently, when it was linked with a single missense mutation in *AIFM1*, encoding mitochondrial apoptosis-inducing factor 1 (AIFM1). This was reported in a Polish family and originally published without MRI findings [6] and in an unrelated Polish family carrying an identical p.(Asp237Gly) mutation [7].

In this study, we present 12 patients with H-SMD, the largest series thus far, all with *AIFM1* variants confined to a locus of approximately 70 bp around the start of exon 7, including synonymous and intronic variants not predicted to be pathogenic by in silico modeling. Thus, we hypothesize that the unique features of this disorder might be caused by tissue-specific loss of functional AIFM1. Variants in *AIFM1* have been shown to be associated with diverse neurological disorders featuring intellectual disability, sensory hearing loss, neuropathy, and an encephalopathy associated with striatal abnormalities on neuroimaging. The present cohort adds to the disease spectrum associated with *AIFM1*, expanding on the single variant previously associated with H-SMD.

## Methods

### Patients

The affected individuals and their families were collected prospectively as part of bioregistries including the Myelin Disorders Bioregistry Project (MDBP) at Children’s National Health System, the Amsterdam Center for Childhood White Matter Disorders and the Yokohama City University Graduate School of Medicine with approval from the institutional review boards at the respective institutions. Written informed consent was obtained for each study participant including specific informed consent for whole-exome sequencing (WES). Genomic DNA samples were collected from blood samples provided to the biorepository. Patient 4 [5] and patients 9–12 [4] were previously published.

### Exome and genome sequencing

WES or whole-genome sequencing (WGS) was performed for individual families to identify causative genes. Exomes were captured using the SeqCap EZ Human Exome Library v.3.0 or SureSelect Human All Exon V5 and sequenced on an Illumina HiSeq 2000 with 100-bp paired-end read-sequencing protocol. Sequencing was performed at the Queensland Centre for Medical Genomics (patient 1), at the Yokohama City University Graduate School of Medicine (patients 2 and 3), at the Department of Genetics, Groningen sequencing center (patient 5–7), and at the University Klinikum Giessen/Marburg (patients 8–12). Two patients (patients 1 and 6) were also analyzed by whole-genome sequencing (WGS), performed by Illumina Inc. in the Illumina Clinical Services Laboratory (San Diego, CA) or in the Illumina United Kingdom sequencing facility in Chesterford, England. Data analysis and variant calling approaches are described in supplemental data. One individual (patient 4) had not consented to next-generation sequencing; therefore, standard Sanger sequencing for *AIFM1* was subsequently performed in view of clinical similarities with other cases. Intron 6 and exon 7 sequences from *AIFM1* across 15 mammalian species were obtained via Ensembl (www.ensembl.org, last accessed on 10 February 2017) to develop sequence logos using WebLogo (http://weblogo.berkeley.edu, last accessed on 10 February 2017).

### Imaging

The latest available MRI was analyzed for this study. MRIs were evaluated according to a previously published protocol [8] for hypomyelination, according to previously defined MRI criteria [9] by N.I.W. and A.V. Clinically obtained bone radiographs were reviewed by D.B. and included skeletal surveys, skull, hand, long bone, hip, and spine films as available.

### Review of clinical findings

Clinical findings were retrospectively collected by review of medical records and testing, and if individuals were available for clinical evaluation, verification of specific findings and features was performed in a prospective fashion.

### AIFM1 expression

#### Cell transdifferentiation/creation of osteoblasts

Transdifferentiation was performed on patient fibroblasts at confluence 90% by adding osteogenic transdifferentiation medium (α-MEM medium (Life Technologies) supplemented with 5 mM β-glycerophosphate (Sigma-Aldrich), 90 μg/ml L-ascorbic acid-2-phosphate (Sigma-Aldrich), 5000 U/ml Penicillin-Streptomycin (Life Technologies), and 5% platelet lysate [8, 10]. After 21 days, Alizarin Red, alkaline phosphatase (ALP) activity, and von Kossa staining were performed to characterize osteogenic properties [9, 11, 12]. The transdifferentiation experiments were repeated three times for every patient cell line except for patient 1 (repeated twice).

#### qPCR

RNA was extracted from transdifferentiated cells on days 2, 3, 7, 14, and 21. qPCR was performed in the Light Cycler® 480 (Roche) for *AIFM1* (NM_004208.3) and *YWHAZ* as housekeeping gene. In order to validate osteogenic transdifferentiation, qPCR of osteoblast markers *RUNX2* and *ALP* was performed (see also supplementary methods). The qPCR results were analyzed with LightCycler 480 software 1.5.1 (Roche) and normalized to housekeeping gene expression. qPCR results were expressed as mean of two independent experiments performed in duplicate for every sample. Differences were analyzed with the use of the paired sample *t* test using GraphPad Prism 6. Differences were considered significant when *p* < 0.05. As cell lines of patient 1 were received later, analysis was performed separately and only qualitatively. Primers are available in supplemental methods.

#### Western blot

Whole cell lysates of fibroblasts and transdifferentiated osteoblasts were subjected to electrophoresis for 50 min at 200 V on NuPage 4–12% BIS-TRIS gel with NuPAGE MOPS Running Buffer (Invitrogen). After protein transfer and blocking, the nitrocellulose membrane was incubated with primary antibodies for AIFM1 (Abcam; cat no. AB1998) and actin (Abcam; cat no. AB 14128) overnight at 4 °C. After 1 h incubation with secondary antibody IRDye 800 CW goat anti-rabbit IgG and the IRDye 680 CW goat anti-mouse IgG antibodies (LICOR Bioscience), the NC membrane was scanned, analyzed, and quantified with Odyssey Infrared Imaging system equipped with the Odyssey v3.0 software (LICOR Bioscience). Additional details are available in supplemental methods.

## Results

### Clinical features

Six unrelated families with 12 affected males with the characteristic combination of spondylometaphyseal dysplasia and CNS hypomyelination were identified (Supplemental Tables 1 and 2). Clinical features were remarkably homogenous. Gestational and perinatal history were uneventful. The children initially had a period of normal development, ranging from 8 to 36 months. In some cases, dysmorphic features were noted during this period, including depressed nasal bridge and midface hypoplasia. Patients never either gained the ability to walk independently or gained independent ambulation with significant delays (14–60 months), sometimes only to lose independent ambulation again within a few years (with the exception of patients 11 and 12 who had normal early milestones). Subsequently, gradual loss of motor function ensued, sometimes after a described plateau or slowing of developmental milestones. Onset of gait abnormalities was between 12 and 24 months (with two exceptions, in whom disease onset was reported at 4.5 and 6 years). Gait abnormalities were due to spasticity, ataxia, proximal weakness, and joint contractures. Cognitive function was mildly impaired in 5/7 individuals for whom data was available. Language was additionally impaired by dysarthria in 6/12 individuals.

Extra-neurologic manifestations including skeletal, eye, and lung abnormalities were present. Patients 10, 11, and 12 were noted to have a shortened nasal bridge. Joint contractures were sometimes noted early in disease course (5/12 at presentation), and enlarged joints were noted in almost all patients (11/12) over time. Postnatal growth failure, with borderline or frank short stature, was noted in all patients where height measurements were available (*n* = 7) and stature was 2–7 SD below the norm, though head circumference was normal. Scoliosis developed in all but one individual for whom this data was available (10/11) and was present from an early age in most. Nystagmus was noted in 10/12 individuals. Vision loss was seen in 8/12 individuals, associated with features of macular degeneration, achromatopsia, optic nerve atrophy and in one individual a rod-cone dystrophy diagnosed on ERG (patient 1). One child (patient 8) had moderate hearing loss. Three individuals in one extended family (patients 5, 6, 7) had pulmonary hypertension. Patient 12 had seizures. No additional extra-neurologic organ involvement was reported.

Seven of the 12 individuals were deceased at the time of this review (ages 35 months to 37 years at time of death). Causes of death included pulmonary hypertension (two children) and aspiration pneumonia. The remaining five are alive (ages 8–18 years old).

### MRI imaging

**Fig. 1:**
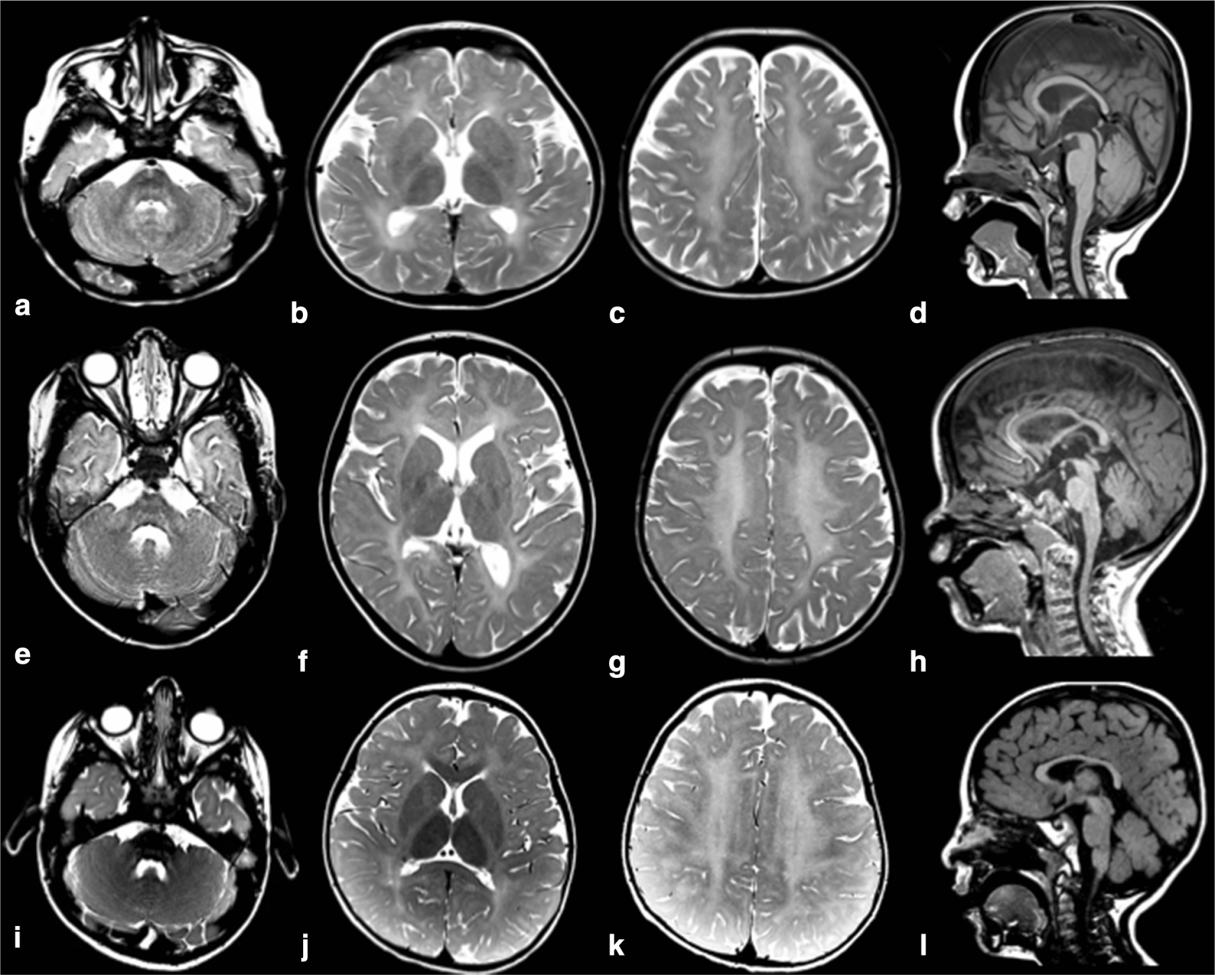
MRI findings in H-SMD. Axial T2-weighted images (**a**–**c**, **e**–**g**, **i**–**k**) and sagittal T1-weighted images (**d**, **h**, **l**) of patient 7 at age 4 years (first row), patient 8 at age 5 years (second row), and patient 1 at age 4 years (third row). The supratentorial white matter shows a diffusely elevated T2 signal (**b**, **c**, **f**, **g**, **j**, **k**), with unremarkable T1 signal (**d**, **h**, **l**) indicating hypomyelination. Signal of the basal ganglia is normal (**b**, **f**, **j**). The cerebellum has a normal volume (**a**, **d**, **e**, **h**, **i**, **l**). Signal of the superior cerebellar peduncles is hyperintense in all patients (**a**, **e**, **i**). Patient 7 has also signal elevation of the hilus of the dentate nucleus (**a**). Patient 8 of the external part of the middle cerebellar peduncle (**e**). Patient 1 has relatively better myelination of the brainstem, corpus callosum and deep gray nuclei

All affected individuals in whom MR imaging was available (9/12) showed hypomyelination, with T1 hyperintense or isointense signal, and T2 hyperintensity diffusely involving the cerebral white matter including the arcuate fibers, the internal capsule, and the corpus callosum (Fig. 1), though in some individuals, these were spared. The basal ganglia, brainstem, and cerebellum were relatively spared, though signal abnormalities were seen in the middle and superior cerebellar peduncles and in some individuals also the hilus of the dentate. There was mild loss of white matter volume and thinning of the corpus callosum, without evident volume loss of basal ganglia and cerebellum. No abnormalities were evident on susceptibility-weighted images or diffusion-weighted images, where available. No contrast enhancement was seen where available.

### Skeletal imaging

**Fig. 2:**
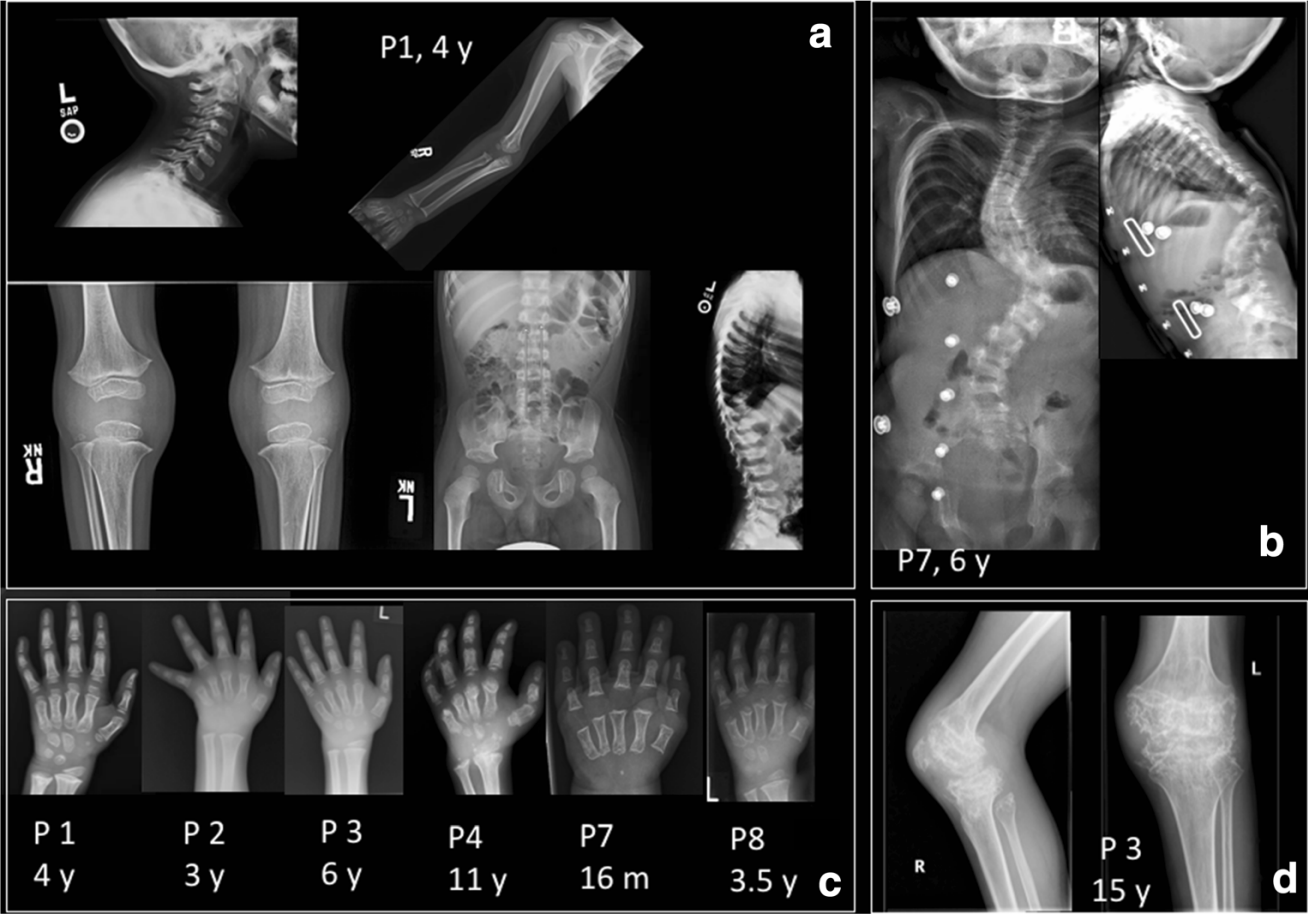
Spondylometaphyseal dyplasia in H-SMD. **a** Common findings in patients included changes in the metaphyses and epiphyses of long bones, including mild flaring, pelvic abnormalities including flat acetabula, squared iliac wings, and thick pubic rami, as well as vertebral abnormalities including end plate irregularity and kyphosis. **b** In some patients over time, vertebral anomalies evolved to include anterior central beaking with vertebra plana, posterior scalloping of lumbar vertebrae, and evolution of severe kyphoscoliosis. **c** In the hands, brachydactyly and clinodactyly with flat, shortened metacarpals and phalanges were universally seen, with variable amounts of coned epiphyses. In older individuals, irregular sclerosis was seen over time. **d** In metaphyses and epiphyses of long bones, severe irregular sclerosis was seen over time, present here at the knees

Patients 1–4 and 7 and 8 had sufficient images for review (Supplemental Table 3). Skeletal abnormalities were present in images collected as early as 16 months but were also progressive and degenerative in nature on images of individuals in the teenage years (Fig. 2). In the hands, brachydactyly and clinodactyly with flat, shortened metacarpals and phalanges were universally seen, with variable amounts of coned epiphyses. In long bones, all individuals had changes in metaphyses and physis, ranging from mild flaring seen early in disease to severe irregular sclerosis at the metaphyses of the long bones, progressive in older patients. In severe cases, epiphyseal irregular sclerosis developed over time at the knees, elbows, and wrists. Vertebral abnormalities were common and included end plate irregularity in milder cases and in more severe cases anterior central beaking with vertebra plana, posterior scalloping of lumbar vertebrae, and evolution of severe kyphoscoliosis. In a few cases, there was narrow interpeduncular distance. The pelvis was notable for flat acetabula, coxa valga or coxa vara, squared iliac wings, thick pubic rami, and progressive sclerosis of femoral epiphyses with coxa magna in some individuals. Diffuse osteopenia was a common finding and present early in most cases.

### Genetic findings

**Fig. 3:**
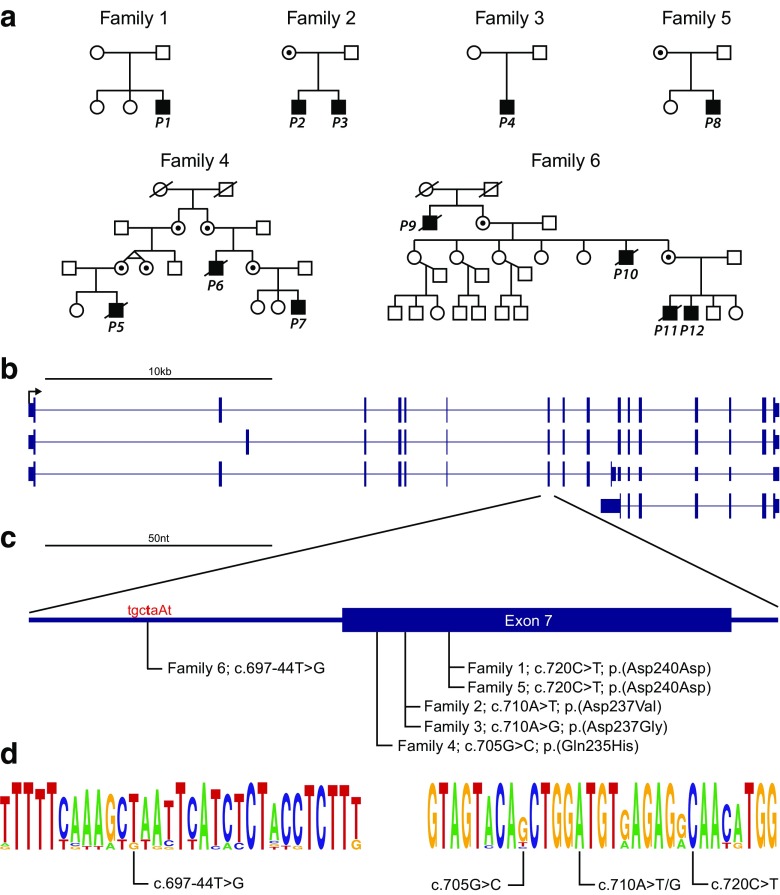
H-SMD-associated mutations in *AIFM1.* **a** Pedigrees of each H-SMD families with subjects of this study indicated. Where the mother is not noted to be a carrier, the mother has been sequenced and the variant is de novo*.* **b** *AIFM1* refseq transcripts showing known alternative splicing events. **c** H-SMD-associated mutations in *AIFM1* are all near exon 7 splice acceptor site. The predicted intron branch point motif is indicated in red text. The Human Splicing Finder tool predicts the c.697-44 T > G mutation will disrupt the function of this branch point. **d** Motifs of primary sequence conservation surrounding each H-SMD-associated mutation in *AIFM1* based on alignment of 15 mammalian species using WebLogo. The size of the letters indicates degree of conservation and where more than one nucleotide is visible suggests variation across species

In all six families, maternally inherited or de novo mutations in or near exon 7 of *AIFM1* were identified based on whole-exome sequencing (Table 1 and Fig. 3). Patient 4 (family 3) has a de novo c.710A > G (p.(Asp237Gly)) mutation identical to the mutation previously reported in two H-SMD families by Mierzewska et al. [7]. Patients 2 and 3 (family 2) had a c.710A > T (p.(Asp237Val)) mutation at the same nucleotide position. Patients 5, 6, and 7, from family 4, carried the missense variant c.705G > C (p.(Gln235His)). Patient 1 (family 1) and patient 8 (family 5) both had the synonymous mutation c.720C > T (p.(Asp240Asp)); this variant was de novo in patient 1 and maternally inherited in patient 8. Patients 9, 10, 11, and 12, all from family 6, had a rare variant 44 bp from the 3′ splice site of exon 7 at position c.697-44 T > G that is predicted to disrupt the intron branch point (Supplemental Table 4) [13]. The carrier status of the mothers of families 1, 2, 3, and 5 were confirmed by Sanger sequencing. The mutations in families 1 and 3 were found to be de novo while family 2 and 5 were maternally inherited. No variant was present in ExAC or dbSNP 147. However, the ExAC database does report a single female individual heterozygous for c.705G > C (p.(Gln235His)), the same nucleotide position and resulting amino acid change seen in family 4.

**Table 1:** Genetic variants in *AIFM1* identified by patient

Family	Base pair	Amino acid	Evidence of pathogenicity [1]	Variant clasification [1]
Family 1 (P1)	c.720C > T	p.(Asp240Asp)	PS2 PS3 PM2	Pathogenic
Family 2 (P2, 3)	c.710A > T	p.(Asp237Val)	PS3 PM2 PM5 [7] PP1 PP3	Pathogenic
Family 3 (P4) [5]	c.710A > G	p.(Asp237Gly) (identical mutation as previously published [7] though in different ethnicity)	PS1 [7] PS2 PS3 PM2 PP3	Pathogenic
Family 4 (P5, 6, 7)	c.705G > C	p.(Gln235His)	PS3 PM2 PP1 (very strong family segregation) PP3	Likely pathogenic
Family 5 (P8)	c.720C > T	p.(Asp240Asp) (unrelated to patient 1)	PS2 PS3 PM2	Pathogenic
Family 6 (P9, 10, 11, 12) [4]	c.697-44 T > G	NA	PS3 PM2 PP1 (very strong family segregation) PP3 (intronic (44 nt 5′ of splice site, disrupts predicted branch point) [13])	Likely pathogenic

Genetic variants in AIFM1 identified by patient with their classification based on Richards et al. 2015 [14]

*PM* pathogenic moderate, *PP* pathogenic supporting, *PS* pathogenic strong, *NA* not available.

The presence in all reported cases of H-SMD of maternally inherited or de novo mutations confined to an approximately 70 bp region of *AIFM1* in and near exon 7, together with a phenotype distinct from previously reported AIFM1 mutations, leads us to suspect these mutations result in loss of functional and stable AIFM1. We used the Human Splicing Finder (v3.0) tool to predict the impact each of the mutations may have on the splicing of AIFM1 [13]. Each of the exonic mutations was predicted to disrupt “Exonic Splicing Enhancer” motifs and potentially result in an alteration of splicing (Supplemental Table 4).

### AIFM1 expression

**Fig. 4:**
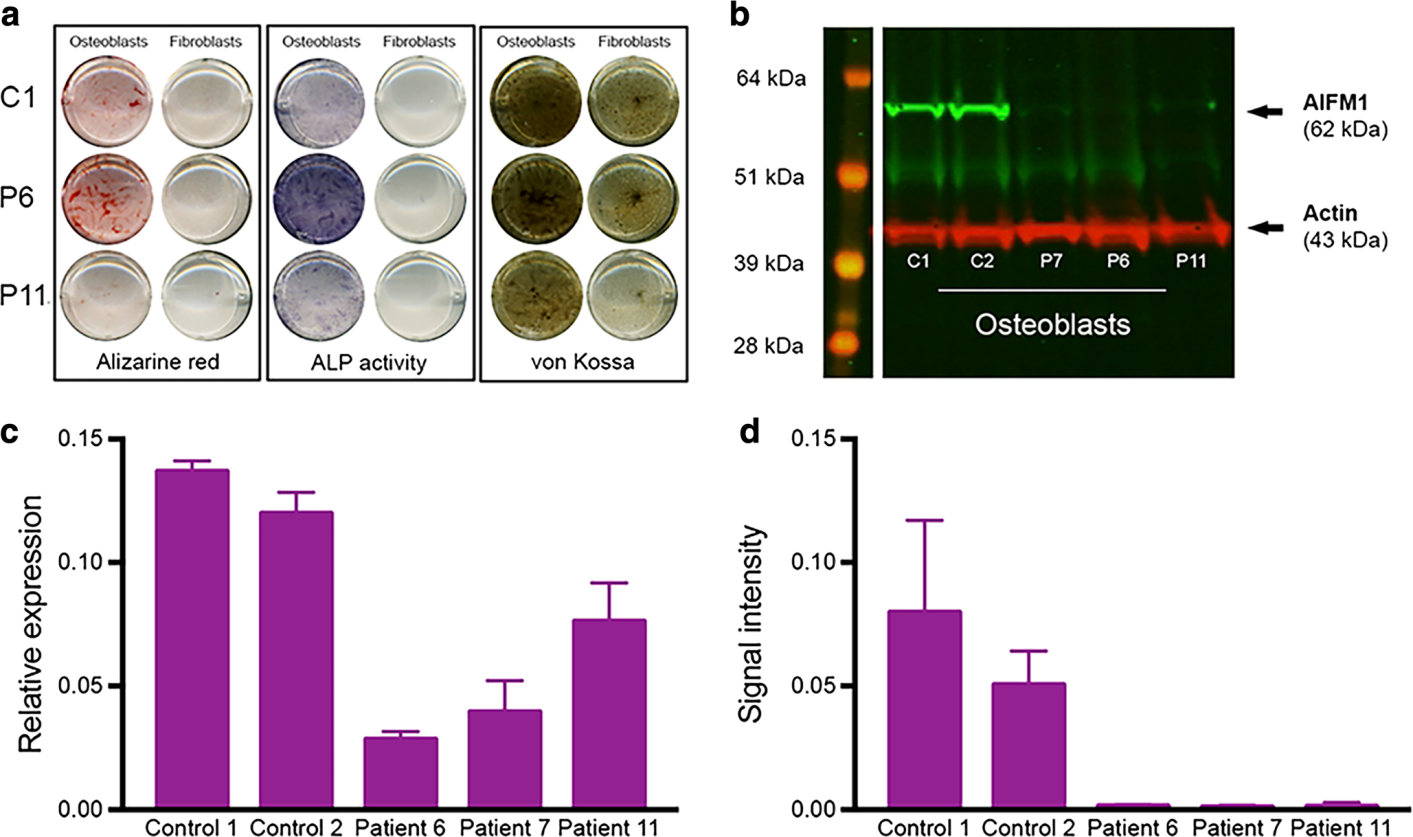
AIFM1 in fibroblasts and transdifferentiated osteoblasts. **a** Alizarin red, alkaline phosphatase (ALP) activity, and von Kossa staining of fibroblasts and transdifferentiated osteoblasts, confirming successful transdifferentiation. These stains provide a qualitative indication of the capability of the cells to mineralize; plates were not normalized for cell number. **b** Western blot of AIFM1 and actin in transdifferentiated osteoblasts (day 21). *kDa* kiloDalton. **c** *AIFM1* relative mRNA expression in transdifferentiated osteoblasts (day 21), in relation to the housekeeping gene *YWHAZ*. **d** Depicts AIFM1 protein expression normalized to Actin in osteoblasts. Error bars indicate standard error of the mean (SEM, *n* = 3)

To investigate the impact of the patient mutations on *AIFM1* expression, we compared *AIFM1* messenger RNA (mRNA) and protein levels in patient fibroblasts and transdifferentiated osteoblasts to equivalent cells from unrelated controls. Osteoblasts were selected in order to obtain cells modeling affected tissue, and transdifferentiation was confirmed by expression of osteogenic markers (*RUNX* and *ALP*, Supplemental Fig. 1) and staining (Fig. 4a). Analysis by quantitative real-time PCR found that *AIFM1* mRNA was significantly decreased in patient osteoblasts (*n* = 3) (*p* = 0.002) when compared to controls (*n* = 3) (Fig. 4c) and also in patient 1 (analyzed, separately; supplementary Fig. 2A). Analysis of osteoblasts by western blot found that AIFM1 protein levels were significantly reduced in patients (*n* = 3) when compared to controls (*n* = 3) (*p* = 0.01) (Fig. 4d); this was confirmed by testing cell lines of a fourth patient in a separate experiment (patient 1, supplementary Fig. 2B). Importantly, the patient cell lines tested included the p.(Gln235His) missense mutation, the p.(Asp240Asp) synonymous mutation, and the c.697-44 T > G intronic branch point mutation indicating that each of these three classes of mutation result in a similar loss of AIFM1 expression in osteoblasts. In fibroblasts, AIFM1 protein expression was very low in controls and undetectable in patients, despite considerable mRNA expression (supplementary Fig. 3).

## Discussion

Here, we show that a rare X-linked condition characterized by hypomyelinating leukodystrophy and spondylometaphyseal dysplasia (H-SMD) is caused by specific *AIFM1* mutations, all located in a small part of the gene comprising about 70 bp around the start of exon 7. The combination of hypomyelination and skeletal dysplasia allows clinical diagnosis and should enable identification of additional patients, although this disorder seems to be extremely rare, because of its rarity both in large cohorts of white matter disease (NIW and AV) and in of skeletal dysplasias (ASF). Neurological course is relatively mild, comparable with 4H syndrome and much milder than in PMD, with a stable phase of variable length followed by slow neurological decline, manifesting in progressive spasticity and ataxia. The prominent skeletal abnormalities also contribute to motor impairment.

Mitochondrial apoptosis-inducing factor 1 (encoded by *AIFM1*, located on Xq26.1) is a protein with multiple canonical roles. AIFM1 has redox activity that aids in oxidative phosphorylation, is proposed to function in the mitochondria to assemble and/or maintain the mitochondrial respiratory complexes I and III, and plays a role in apoptosis, triggering caspase-independent programmed cell death under apoptotic stimuli [15–17].

Why this specific phenotype with its characteristic combination of myelin deficit and bone abnormalities is caused by this subset of *AIFM1* mutations is not known. In a recent study on the effect of several *AIFM1* mutations on protein structure and function, one mutation situated in close vicinity to the mutations described here, p.(Val243Leu), had virtually no effect on the folding, redox, and DNA-binding properties of the protein [18]. Even more intriguing, different variants in *AIFM1* have previously been shown to be associated with diverse neurological disorders featuring intellectual disability, sensory hearing loss, neuropathy, and T2 hyperintensity in the striatum on magnetic resonance imaging (MRI), with some patients presenting with a mitochondrial phenotype and abnormalities in respiratory chain function [19]. Specific phenotypes include auditory neuropathy spectrum disorder [20], a severe X-linked mitochondrial encephalopathy [21, 22], and Cowchock syndrome, an axonal sensorimotor neuropathy with deafness and mental retardation [23, 24]. This suggests that there is a tight genotype-phenotype relationship, causing these different clinical manifestations.

The specific clinical presentation of H-SMD is clearly distinct from other conditions previously associated with mutations in *AIFM1*. Given all known cases of H-SMD have a single base mutation within a 70-nt region flanking the exon 7 acceptor splice site, we suspect that mutations in this specific region of *AIFM1* result in a common functional consequence that specifically gives rise to H-SMD. Three of the five known H-SMD-associated AIFM1 mutations are missense, and it is possible that the encoded amino acid changes may result in disrupted *AIFM1* protein function. However, as three H-SMD families have mutations in this region that do not directly alter the encoded protein sequence, it is most likely that the primary functional consequence of all five known H-SMD mutations (including missense, synonymous, and intronic) is a specific defect in mRNA splicing. This hypothesis is consistent with our findings that *AIFM1* mRNA levels are significantly reduced in patient-derived osteoblast cells and AIFM1 protein levels reduced to near the limits of detection. We did attempt to detect evidence of a specific alteration in *AIFM1* splicing by quantitative real-time PCR using a series of exon/exon specific primer pairs across the gene. However, we could not detect any definitive changes between patient and control cell lines. Thus, although the association of these mutations within a small genomic area is compelling, the precise mechanism by which they disrupt AIFM1 function is still unclear.

H-SMD is not the only disorder with spondylometaphyseal involvement and associated neurological symptoms. One example is *TRPV4*-related disorders. Mutations in *TRPV4*, encoding a calcium-permeable cation channel, give rise to either skeletal dysplasia, sometimes with early lethality, or peripheral neuropathy or a combination of both [25–27]. Neither the pathogenic mechanisms leading to these diverse phenotypes affecting completely different tissues nor the effects of specific mutations are yet understood. The example of H-SMD demonstrates how significant genotype-phenotype correlations may be in certain genes and emphasizes the importance of tissue-specific impacts of gene mutations.

## Electronic supplementary material

ESM 1 (PDF 7902 kb)
