# Supplementary material for: X-linked hypomyelination with spondylometaphyseal dysplasia (H-SMD) associated with mutations in AIFM1
Source: Neurogenetics. 2017 Aug 26;18(4):185–94. doi: 10.1007/s10048-017-0520-x (PMC5705759; doi:10.1007/s10048-017-0520-x)
Supplement: Supplementary file 1 — (PDF 7902 kb) [file 10048_2017_520_MOESM1_ESM.pdf]

# X-linked Hypomyelination with Spondylometaphyseal Dysplasia (H-SMD) associated with mutations in *AIFM1*

Noriko Miyake[1*], Nicole I. Wolf[2*, ***], Ferdy K. Cayami[2,3,4*], Stephen J Bent[5], Annette Bley[6], Dorothy Bulas[7], Alex Conant[8], Joanna Crawford[5], Karen W Gripp[9], Andreas Hahn[10], Sean Humphray[11], Shihoko Kimura-Ohba[12], Zoya Kingsbury[11], Bryan R. Lajoie[13], Dennis Lal[14], Dimitra Micha[3], Amy Pizzino[8], Richard J Sinke[15], Deborah Sival[16], Irene Stolte-Dijkstra[15], Andrea Superti-Furga[17], Nicole Ulrick[8], Ryan J Taft[5,13, 18], Tsutomu Ogata[19], Keiichi Ozono[12], Naomichi Matsumoto[1], Bernd A. Neubauer [10**], Cas Simons[5**], Adeline Vanderver[8, 17, 20**]

1. Department of Human Genetics, Yokohama City University Graduate School of Medicine, Fukuura, Kanazawa-kuYokohama 236-0004, Japan;(nmiyake@yokohama-cu.ac.jp, naomat@yokohama-cu.ac.jp)
2. Department of Child Neurology, and Amsterdam Neuroscience, VU University Medical Center, De Boelelaan 1117, 1081 HV Amsterdam, the Netherlands (n.wolf@vumc.nl; f.cayami@vumc.nl, ms.vanderknaap@vumc.nl)
3. Department of Clinical Genetics, VU University Medical Center, De Boelelaan 1117, 1081 HV Amsterdam, the Netherlands (d.micha@vumc.nl)
4. Center for Biomedical Research, Faculty of Medicine, Diponegoro University, Semarang, Indonesia (f.cayami@vumc.nl)
5. Institute for Molecular Bioscience, The University of Queensland, Brisbane, Australia, c.simons@uq.edu.au, +61-7-334-62080 (c.simons@uq.edu.au, j.crawford@imb.uq.edu.au, s.bent@imb.uq.edu.au)
6. University Children's Hospital, University Medical Center Hamburg Eppendorf, Martinistr. 52, 20246 Hamburg, Germany (abley@uke.uni-hamburg.de)
7. Children's National Medical Center, Department of Diagnostic Imaging and Radiology, Washington DC, United States, (dbulas@childrensnational.org)
8. Children's National Medical Center, Department of Neurology, Suite 4800, Washington DC, United States, avanderver@childrensnational.org, 202-476-4959 (avanderv@childrensnational.org, nulrick@childrensnational.org, apizzino@childrensnational.org, aconant@childrensnational.org)
9. Division of Medical Genetics, A.I. duPont Hospital for Children/Nemours, Wilmington, DE, USA (kgripp@Nemours.org)
10. Univ.-Klinikum Giessen / Marburg; Standort Giessen, Department of Pediatric Neurology, Feulgenstr. 12, 35389 Giessen, Germany (Andreas.Hahn@paediat.med.uni-giessen.de, Bernd.A.Neubauer@paediat.med.uni-giessen.de)
11. Illumina, Inc., Chesterford Research Park, Little Chesterford CB10 1XL, UK. (shumphray@illumina.com, zkingsbury@illumina.com)
12. Department of Pediatrics, Osaka University Graduate School of Medicine, Osaka, Japan, keioz@ped.med.osaka-u.ac.jp; skimura@ped.med.osaka-u.ac.jp
13. Illumina, Inc, San Diego, CA USA (rtaft@illumina.com)
14. Psychiatric and Neurodevelopmental Genetics Unit, Massachusetts General Hospital and Harvard Medical School, Boston, Massachusetts; and Stanley Center for Psychiatric Research, Broad Institute United States of America dlal@broadinstitute.org
15. Department of Genetics, University Medical Center Groningen, University of Groningen, Groningen, The Netherlands r.j.sinke@umcg.nl, i.stolte@umcg.nl
16. Department of Child Neurology, University Hospital Groningen, Netherlands d.a.sival@umcg.nl,
17. Division of Genetic Medicine, Centre Hospitalier Universitaire Vaudois (CHUV), University of Lausanne, Lausanne, Switzerland email: asuperti@unil.ch
18. George Washington University School of Medicine, Washington DC, United States
19. Department of Pediatrics, Hamamatsu University School of Medicine, Hamamatsu, 431-3192, Japan (tomogata@hama-med.ac.jp).
20. Children's Hospital of Philadelphia, Philadelphia, PA, USA

** shared first, ** shared last, *** corresponding*

## Supplemental Methods

*Whole exome sequencing (WES)*

Exomes were captured using the SeqCap EZ Human Exome Library v.3.0 SureSelect Human All Exon V5, and sequenced on an Illumina HiSeq 2000 with 100 bp paired-end read-sequencing protocol in different places for different patients: This was done at the Queensland Centre for Medical Genomics (patient 1); the Yokohama City University Graduate School of Medicine (patients 2 and 3); at the Department of Genetics, Groningen Sequencing Center (patient 5 – 7); the University Klinikum Giessen / Marburg (patients 8 – 12). Two patients (patients 1 and 6) were also analysed by whole genome sequencing (WGS), performed by Illumina Inc in the Illumina Clinical Services Laboratory (San Diego, CA) or in the Illumina United Kingdom sequencing facility in Chesterford, England.. Reads were aligned to the reference human genome (UCSC Genome Browser hg19) with Burrows-Wheeler Aligner (BWA) and downstream processing of data was done with Genome Analysis Toolkit (GATK) v.2.2.8, Picard v.1.8, and SAMtools v.0.1.18. Variants (SNPs and indels) were identified with GATK according to version four of the GATK Best Practice Variant Detection guide or Varscan v.2.2.5. Variants were annotated with the use of Annovar with UCSC Known Genes models, and known polymorphisms were identified with dbSNP135, 1000 Genomes (April 30, 2012, release) and the National Heart, Lung, and Blood Institute (NHLBI) Exome Sequencing Project (ESP) Exome Variant Server (ESP6500 release). Minor allele frequencies were recorded from each data set. Subsequent analysis and identification of candidate variants was performed with an in-house workflow incorporating the annotated variant data and pedigree information. Variant analysis for the patients 2 and 3 was performed at the Yokohama City University Graduate School of Medicine.[1]

Cell transdifferentiation/creation of osteoblasts: Three days prior to transdifferentiation, fibroblasts were seeded in 12 well-plates at a density of $1x10^6$ cells per well. Transdifferentiation was started when confluence reached 90% by adding osteogenic transdifferentiation medium (α-MEM medium (Life Technologies) supplemented with 5 mM β-glycerolphosphate (Sigma-Aldrich), 90 µg/ml L-ascorbic acid-2-phospate (Sigma-Aldrich), 5000 U/ml Penicillin-Streptomycin (Life Technologies) and 5% platelet lysate) at 37ºC and 5% $CO_2$. Platelet lysate were prepared as previously described.[2; 3] Cells were cultured for 21 days with replacement of freshly prepared medium twice per week. After 21 days, Alizarin Red, alkaline phosphatase (ALP) activity and von Kossa staining was performed to characterize osteogenic properties.

Cycloheximide treatment: To detect the effect of the mutation on splicing, RNA from primary fibroblasts treated with and without cyclohemixide for 4.5 hours was isolated using RNA isolation kit Quick-RNA® MiniPrep (Zymo Research) according to the manufacturer's instructions. After measuring quality and quantity of RNA with Nanodrop® 1000 (Nanodrop), cDNA synthesis was performed using Superscript II Reverse Transcriptase. The amplification for PCR was done with Light Cycler SYBR Green I Master (Roche) for 45 cycles of 10 seconds at 95ºC for denaturation, 20 seconds at 56ºC for annealing and 30 seconds at 72ºC for extension with following primers: 5'-TGCCTGCTGCTCCTTTACTTC-3' and 5'-TAACCCCCTCTCGTCTGACTTT-3'(*AIFM1*, NM_004208.3). PCR products were analysed on 2% agarose gel.

qPCR: RNA was extracted from fibroblasts and transdifferentiated cells on days 2, 3, 7, 14 and 21 with Quick RNA mini prep kit (Zymo Research) according to the manufacturer's protocol. RNA quality and quantity were measured with NanoDrop®

1000 (NanoDrop). cDNA was synthesized with SuperScript® II Reverse Transcriptase (Invitrogen). qPCR was performed in Light Cycler® 480 (Roche) for *AIFM1* (NM_004208.3) (forward primer 5'– TTGAGAATGGTGGTGTGGCT – 3' and reverse primer 5'– AGACTTCTTGGAGTACCTCCTGT – 3') and *YWHAZ* as housekeeping gene (forward primer 5'– GATGAAGCCATTGCTGAACTTG – 3' and reverse primer 5'– CTATTTGTGGGACAGCATGGA – 3'). In order to validate osteogenic transdifferentiation, qCPR of osteoblast markers *RUNX2* and *ALP* was performed with primers as follow: *RUNX2* forward: 5' – ATGCTTCATTCGCCTCAC – 3' ; reverse: 5' – ACTGCTTGCAGCCTTAAAT – 3'; *ALP* forward: 5' – AGGGACATTGACGTGATCAT – 3'; reverse: 5' – CCTGGCTCGAAGAGACC – 3'. Amplification of PCR products was carried out with a preamplification step of 10 minutes in 95ºC followed by 45 cycles of 10 s in 95ºC, 5s in 56ºC and 10s in 72ºC. The qPCR results were analyzed with LightCycler 480 software 1.5.1 (Roche) and normalized to the housekeeping gene expression. qPCRs were done in duplicate and repeated twice for all samples.

<u>Western blot:</u> Whole cell lysates of fibroblasts and transdifferentiated osteoblasts were prepared after 21 days using 4X NuPAGE® LDS Sample Buffer (Invitrogen) and NuPage Sample reducing agent (Invitrogen). After incubation in 95$^{0}$C for 10 minutes, protein lysates were subjected to electrophoresis for 50 minutes in 200V on NuPage 4-12% BIS-TRIS gel with NuPAGE MOPS Running Buffer (Invitrogen). After transferring protein to nitrocellulose membrane with iBlot® Transfer stack (Invitrogen) and blocking with Odyssey blocking buffer (LICOR Bioscience), the NC membrane was incubated with primary antibodies for AIFM1 (Abcam; Cat No. AB1998) and Actin (Abcam; Cat no.AB 14128) overnight at 4$^{0}$C. After 1 hour incubation with secondary antibody IRDye 800 CW goat anti-rabbit IgG and the IRDye 680 CW goat

anti-mouse IgG antibodies (LICOR Bioscience), the NC membrane was scanned, analyzed and quantified with Odyssey Infrared Imaging system equipped with the Odyssey v3.0 software (LICOR Bioscience).

## Supplemental Table 1. Clinical Characteristics

| Patient | 1 | 2 | 3 | 4 | 5 | 6 | 7 | 8 | 9 | 10 | 11 | 12 |
|---|---|---|---|---|---|---|---|---|---|---|---|---|
| Gender | Male | Male | Male | Male | Male | Male | Male | Male | Male | Male | Male | Male |
| Year of birth | 2009 | 1997 | 1999 | 1992 | 2006 | 1975 | 2007 | 2007 | 1940 | 1961 | 1985 | 1987 |
| Year of death | - | - | - | 2012 | 2009 | 1980 | - | - | 1966 | 1998 | 2004 | NA |
| Siblings* | -/2F | + 1M/- | + 1M/- | -/- | - | - / F | - / 2F | - / F | -/1F | -/6F | +1M/-1M/1F | +1M/-1M/1F |
| **Past History** | | | | | | | | | | | | |
| Gestation | Normal | Normal | Normal | Normal | Normal | Normal | Normal | Normal | Normal | Normal | Normal | Normal |
| Birth length | 53 cm | 45 cm | 47 cm | NA | 43 cm | 48 cm | 48 cm | Normal | NA | NA | Normal | Normal |
| Birth weight | 3820g | 2885 g | 3170 g | 3088g | 2815 g | 2600 g | 2465 g | Normal | NA | NA | Normal | Normal |
| Initial development | Normal until 14 mo | Normal until 1.5 y | Normal until 21 mo | Normal until 10 mo | Normal | Normal | Normal | Delayed motor skills | Normal | Normal | Normal | Normal |
| **Presentation** | | | | | | | | | | | | |
| Age at onset | 16 mo | 21 mo | 54 mo | 12 mo | 12 mo | 24 mo | 12 mo | 8 mo | 6 y | 2-3 y | 3 y | 3 y |
| Signs at presentation | Abnormal gait | Joint contractures , abnormal gait, short stature | Abnormal gait, joint contracture | Joint contractures , Motor delay | Growth and motor delay Dysmorphic | Growth delay Dysmorphic | Growth delay, Dysmorphic | Delayed gross motor function, enlarged joints | Spastic diplegia, tremor, reduced vision, enlarged joints | Spastic diplegia, tremor, reduced vision, enlarged joints | Spastic diplegia, tremor, reduced vision, enlarged joints | Spastic diplegia, tremor, reduced vision, enlarged joints |
| **Course over time** | | | | | | | | | | | | |
| Nystagmus (age) | + (22 mo) | - | - | + | + | + | + (5.5 y) | +(2 y) | + (6-7 y) | + (6 y) | + | + |
| Retinopathy or other (age) | + (48 mo) | Optic nerve atrophy, macular changes and other | Optic nerve atrophy, macular changes and other | Optic nerve atrophy (+) | - | + | - | - | - | + (Post mortem) | - | - |
| Speech changes (age) | +(48 mo) | - | - | + (5 or 6 y) | - | - | + (5 y) | - | NA | NA | + (6 y) | +(6 y) |
| Short stature (first noticed) | + (28 mo) | + (3 y) | + (4 y) | + (18 mo) | + (1 y) | + (2 y) | + (1 y) | + | NA | NA | + | + |
| Enlarged joints (first noticed) | + (28 mo) | + (NA) | + (10y) | + | - | + | + (6 y) | + (at onset) | + (at onset) | + (at onset) | + (at onset) | + (at onset) |
| Scoliosis (diagnosed at age) | + (30 mo) | + (24 mo) | + | + | + (2 y 8 mo) | + (4 y) | + (2 y) | + (7 y) | NA | + (36 y) | - | + (27 y) |
| Cardiomyopathy | - | - | - | - | - pulm HT + | - pulm HT + | - pulm HT | - | NA | NA | - | - |
| Gastric tube (at age) | - | - | - | - | + (2 y) | - | + (6 y) | - | NA | NA | NA | + (27 y) |

| | | | | | | | | | | | | |
|---|---|---|---|---|---|---|---|---|---|---|---|---|
| Regression | + (4.5 y) | + | + | + (before 2 y) | + | + (after 4 y) | + | + (3-4 y) | + (6 y) | + (6 y) | + (5 y) | + (5 y) |
| Unsupported walking (months) | 22 mo | 18 mo | 21 mo | Never walked | 25 mo | 18 mo | 5 y | - | NA | NA | 14 mo | 14 mo |
| Loss of unsupported walking (years) | 42 mo | - | - | Never walked | 27 mo | 4y | NA | NA | NA | 16 y | 16 y | 16 y |
| Present cognitive level | Normal | Mild MR | Normal | Mild MR | NA | NA | Mild MR | Mild MR | NA | NA | Mild MR | NA |
| Present speech/communication (by CFCS or other) | II | Within normal range | Within normal range | Language-social 55 points (average 100) at 9 y | NA | NA | II | II | NA | NA | NA | Until 2014: II-IV, subsequent tracheostomy |
| Present motor level (by GMFCS) | III | III | IV | III at 11 y | NA | NA | III | III | NA | NA | NA | Until 2014: III, since 2015: IV |
| **Age at death** | NA | NA | NA | 20 y | 2 y 11 mo | 5 y 11 mo | NA | NA | 26 y | 36 y | 19 y | NA |
| **Cause of death** | NA | NA | NA | Respiratory failure due to infection | Pulm HT | Pulm HT | NA | NA | Aspiration pneumonia | Aspiration pneumonia | Suicide | NA |
| **Symptoms in the mother** | | | | | | | | | | | | |
| Mother carrier | No | Yes | Yes | No | Yes | Yes | Yes | Yes | NA | Yes | Yes | Yes |
| Mother's height (cm, SD) | NA | 148 cm (-2 SD) | 148 cm (-2 SD) | NA | 152 (-2.8 SD) | 158 (-1.9 SD) | 157 (-2.0 SD) | 166 cm (+ 0.5 SD) | NA | NA | NA | NA |

Legend: *Siblings: affected (F or M) / unaffected (F or M); Patient 1 identified by A Vanderver, Patients 2 and 3 identified by N. Matsumoto, Patient 4 previously published in Kimura-Ohba et al., Patients 5, 6, and 7 identified by D. Sival, I. Stolte-Dijkstra, and RJ Sinke, Patient 8 identified by NI Wolf, and Patients 9,10,11 and 12 previously published in Neubauer et al. (corresponding to patients, II-6, III-1 IV-8 and IV-9 respectively). CFCS: communication functional classification system; EMG; electromyography; F: female; GMFCS: gross motor functional classification system; M: male; Mo: month; MR: mental retardation; NA: not applicable or not available; OFC: occipitofrontal head circumference; pulm HT: pulmonary hypertension; SD: standard deviation; Y: year.

## Supplemental Table 2. Physical examination

| Patient | 1 | 2 | 3 | 4 | 5 | 6 | 7 | 8 | 9 | 10 | 11 | 12 |
|---|---|---|---|---|---|---|---|---|---|---|---|---|
| **Last examination** | | | | | | | | | | | | |
| Age (years) | 6.5 y | 18 y | 16 y | 11 y | 2y 10mo | 5 y | 8 y 4 mo | 9 y | 26 y | 36 y | 15 y | 28 y |
| Height (cm, SD) | 102 at 5.5y (-2.3SD) | 134 (-6.3 SD) | 133 (-6.2 SD) | 107 (-5.5 SD) | 77 (-5.4 SD) | 75 at 2 y (-4.1 SD) | 88 (-7.8 SD) | NA but below 3% | NA | NA | 165 (-1.5 SD) | 165 (-1.5 SD) |
| OFC (cm, SD) | 49.5 (-2 SD) | 55.3 (-0.5 SD) | 54.0 (-1 SD) | NA | 52 (+1 SD) | 49.5 at 2 y (mean) | 51 (-1 SD) | NA but normal | NA | NA | NA but normal | NA but normal |
| Language | Normal | Normal | Normal | Normal | Normal | Normal | Normal | Normal | Normal | Normal | Normal | Normal |
| Eyes, vision | Vision loss, retinopathy | Vision loss, retinopathy, optic nerve atrophy, corneal opacity | Vision loss, retinopathy, optic nerve atrophy, corneal opacity | Vision loss, bilateral optic atrophy | NA | NA | Vision loss | Vision loss | NA | NA | Vision loss at 11 y | Severe visual impairement |
| Eye movements | Nystagmus | Normal | Normal | Nystagmus | Nystagmus | Nystagmus | Nystagmus | Nystagmus | Nystagmus | Nystagmus | Nystagmus | Nystagmus |
| Head titubation | - | - | - | - | - | - | - | + (7 y) | NA | + (14 y) | + (14 y) | + (14 y) |
| Hearing | Normal by clinical examination | Normal by clinical examination | Normal by clinical examination | Impaired by BAER at age 11 | Normal by clinical examination | Normal by clinical examination | Normal by clinical examination | Impaired (age 7 years, hearing aids) | Normal by clinical examination | Normal by clinical examination | Normal by clinical examination | Normal by clinical examination |
| Speech | Dysarthria | Normal | Normal | Dysarthria | Normal | Normal | Speech normal, but hoarse voice | Dysarthria | NA | Dysarthria | Dysarthria | Dysarthria |
| Arms | | | | | | | | | | | | |
| Tone | Mild increase | Normal | Normal | Normal | NA | NA | Reduced | Normal | Normal | Normal | Normal | Normal |
| Muscle strength | Reduced | Normal | Normal | Normal | NA | NA | Reduced | Reduced | Normal | Normal | Normal | Reduced |
| Reflexes | Present | Reduced | Reduced | Reduced | NA | NA | Brisk | Normal | Normal | Brisk | Brisk | Brisk |
| Spasticity | + | - | - | + | | | | Mild | Normal | + | + | + |
| Ataxia | + | - | - | + | NA | NA | - | - | Normal | - | - | - |
| Extra-pyramidal signs | - | - | - | NA | NA | NA | - | - | Normal | - | - | - |
| Sensory function | Normal | - | - | NA | NA | NA | - | Normal | Normal | Normal | Normal | Normal |
| Legs | | | | | NA | NA | Normal | | | | | |

| | | | | | | | | | | | | |
|---|---|---|---|---|---|---|---|---|---|---|---|---|
| Tone | Significant increase | Normal | Normal | Normal | NA | NA | Normal | Increased | NA | Increased | Increased | Increased |
| Muscle strength | Proximal weakness | Reduced | Reduced | Reduced | NA | NA | Reduced | Reduced | Reduced | Reduced | Reduced | Reduced |
| Reflexes | Reduced | Reduced | Reduced | Absent | NA | NA | Increased | Brisk | Brisk | Brisk | Brisk | Brisk |
| Babinski signs | + | + (bilateral) | + (bilateral) | + | NA | NA | - | + | + | + | + | + |
| Spasticity | + | - | - | + | NA | NA | Mild | + | + | + | + | + |
| Ataxia | + | - | - | NA | NA | NA | + | + | + | + | + | + |
| Extrapyramidal signs | - | - | - | NA | NA | NA | - | - | - | - | - | - |
| Sensory function | Normal | - | - | NA | NA | NA | normal | Normal | NA | Normal | Normal | Normal |
| Gait | Walker | Cane | Cane, wheelchair | Never walked | NA | Walker | Walker | Never walked | Spastic diplegia | Spastic diplegia | Spastic diplegia | Spastic diplegia |
| Scoliosis | + | + | + | + | + | + | + | Mild | + | + | None | + (27 y) |
| Enlarged joints | + | + | + | + | NA | + | + (knees and wrists) | + (knees and wrists) | + (knees and wrists) | + (knees and wrists) | + (knees and wrists) | + (knees and wrists) |
| Electro-physiologic studies | EMG normal | EMG/NCS nl at 12 years | Not examined | EMG normal | NA | EMG normal | NA | Mixed motor and sensory neuropathy | NA | Normal | Normal | Normal |

Legend: *Siblings: affected (F or M) / unaffected (F or M); Patient 1 identified by A Vanderver, Patients 2 and 3 identified by N. Matsumoto, Patient 4 previously published in Kimura-Ohba et al., Patients 5, 6, and 7 identified by D. Sival, I. Stolte-Dijkstra, and RJ Sinke, Patient 8 identified by NI Wolf, and Patients 9,10,11 and 12 previously published in Neubauer et al. (corresponding to patients IV-9, II-6, IV-8 and III-1 respectively). BAER: brainstem auditory evoked response; CFCS: communication functional classification system; EMG; electromyography; F: female; GMFCS: gross motor functional classification system; M: male; Mo: month; MR: mental retardation; NA: not applicable or not available; NCS: nerve conduction studies; OFC: occipitofrontal head circumference; pulm HT: pulmonary hypertension; SD: standard deviation; Y: year.

# Supplemental table 3. Skeletal characteristics

| Patients | 1 | 2 | 3 | 4 | 7 | 8 |
|---|---|---|---|---|---|---|
| Age of most recent images | 6 y | 15 y | 15 y | 11y | 3.5 y | 6y |
| Skull | | | | | | |
| Brachycephaly | + | + | + | + | + | + |
| Hands | | | | | | |
| Brachdactyly | + (metacarpals) | + (phalanges and metacarpals) | + (phalanges and metacarpals) | + (phalanges and metacarpals) | +(phalanges and metacarpals) | +(phalanges and metacarpals) |
| Clinodactyly | + | + | + | + | + | - |
| Coned epiphyses | + | + (by three years) | - | + with sclerosis of phalanges and metacarpals at young age) | + | - |
| Long bones | | | | | | |
| Metaphyseal flaring in the long bones | ++ (knee, elbow) | ++ (even at 23 mo with progressive tibia vera) | ++ (knee with progressive tibia valga, not radius and ulna) | + | + | NA |
| Epiphyses irregular sclerosis | - | ++ (15 y) | ++ | - | - | NA |
| Metaphyseal sclerosis | + | ++ (even at 23 mo) | ++ | - | - | NA |
| Vertebral abnormalities of cervical spine | | | | | | |
| Vertebral abnormalities | Vertebra planae | NA (no lateral spine) | Nl | Nl | NA | Unable to assess due to scoliosis |
| Vertebral abnormalities of the thoraco lumbar spine | | | | | | |
| End plate irregularity | + | NA (no lateral spine) | + | + | + | Unable to assess due to scoliosis |
| Vertebra planae | + | + (on AP only) | - | + (limited) | - | Unable to assess due to scoliosis |
| Anterior central beaking | + | NA (no lateral spine) | + | + (limited) | - | + (likely gibbus or central beak) |
| Scoliosis | Kyphosis only | + (thoracolumbar scoliosis) | +(thoracolumbar scoliosis) | Lumbar lordosis | Kyphosis only | Severe kyphoscoliosis |
| Narrow interpeduncular distance | + | NA | NA | NA | NA | + |
| Posterior scalloping of lumbar vertebrae | + | NA (no lateral spine) | - | + | + | - |
| Ribs | Nl | Nl | Nl | Nl | Nl | Nl |
| Pelvis | | | | | | |

| | | | | | | |
|---|---|---|---|---|---|---|
| Flat acetabulum | + | - | - | + | NA | - |
| Coxa valga/vara | + (valga) | + (vara with progressive Coxa magna and prominent trochanters) | + (vara with progressive Coxa magna and prominent trochanters) | + (valga) | NA | + (valga) |
| Hip subluxation | + | + | + | + | NA | - |
| Squared iliac wings | + | NA | + | + | NA | - |
| Thick pubic rami | + | NA | + | + | NA | - |
| Progressive sclerosis and flattening of epiphyses | + | + | + | - | NA | - |
| Narrow sacrosciatic notch | - | - | - | + | NA | - |
| Osteopenia | + | + | + | + | + | + |

## Supplemental table 4: Summary of splicing motifs disrupted by AIFM1 mutations predicted by Human Splicing Finder v3.0 tool.

| Predicted signal[1] | Algorithm | Position | Interpretation[1] |
|---|---|---|---|
| **c.697-44T>G** | | | |
| WT branch point broken | HSF matrices | 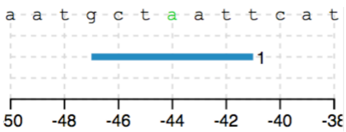  | Alteration of WT Branch Point. Potential alteration of splicing |
| **c.705G>C** | | | |
| ESE Site Broken | 1 - ESE-Finder - SRp40<br>2 - ESE-Finder - SRp55<br>3 - PESE Octamers from Zhang & Chasin | 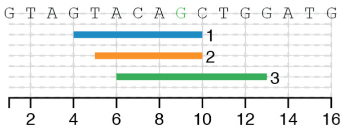  | Alteration of an exonic ESE site. Potential alteration of splicing. |
| **c.710A>T** | | | |
| New Donor Site | 1 - HSF Matrices | 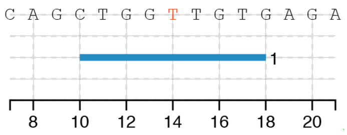  | Activation of an exonic cryptic donor site. Potential alteration of splicing. |
| New ESS Site | 1 - IIEs from Zhang et al.<br>2 - ESR Sequences from Goren et al. | 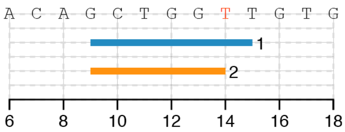  | Creation of an exonic ESS site. Potential alteration of splicing. |
| ESE Site Broken | 1 - PESE Octamers from Zhang & Chasin<br>2 - EIEs from Zhang et al.<br>3 - HSF Matrices - 9G8 | 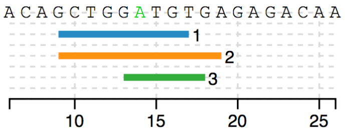  | Potential alteration of splicing. |
| **c.710A>G** | | | |
| New Donor Site | 1 - HSF Matrices | 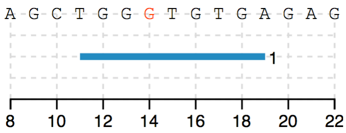  | Activation of an exonic cryptic donor site. Potential alteration of splicing. |
| New ESS Site | 1 - IIEs from Zhang et al.<br>2 - ESR Sequences from Goren et al. | 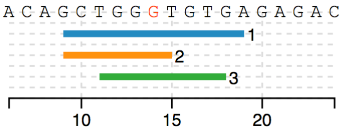  | Creation of an exonic ESS site. Potential alteration of splicing. |
| ESE Site Broken | 1 - PESE Octamers from Zhang & Chasin<br>2 - EIEs from Zhang et al.<br>3 - HSF Matrices - 9G8 | 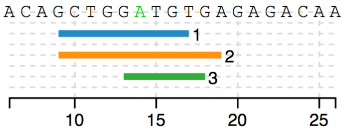  | Potential alteration of splicing. |
| **c.720C>T** | | | |
| ESE Site Broken | 1 - HSF Matrices - 9G8<br>2 - ESE-Finder - SF2/ASF<br>3 - EIEs from Zhang et al.<br>4 - ESE-Finder - SRp40<br>5 - RESCUE ESE Hexamers<br>6 - ESR Sequences from Goren et al. | 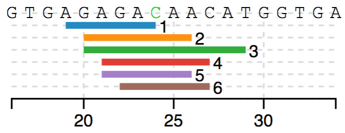  | Alteration of an exonic ESE site. Potential alteration of splicing. |

[1] ESE: Exonic Splicing Enhancers; ESS: Exonic Splicing Silencers.

Figures

## Supplemental Figure 1

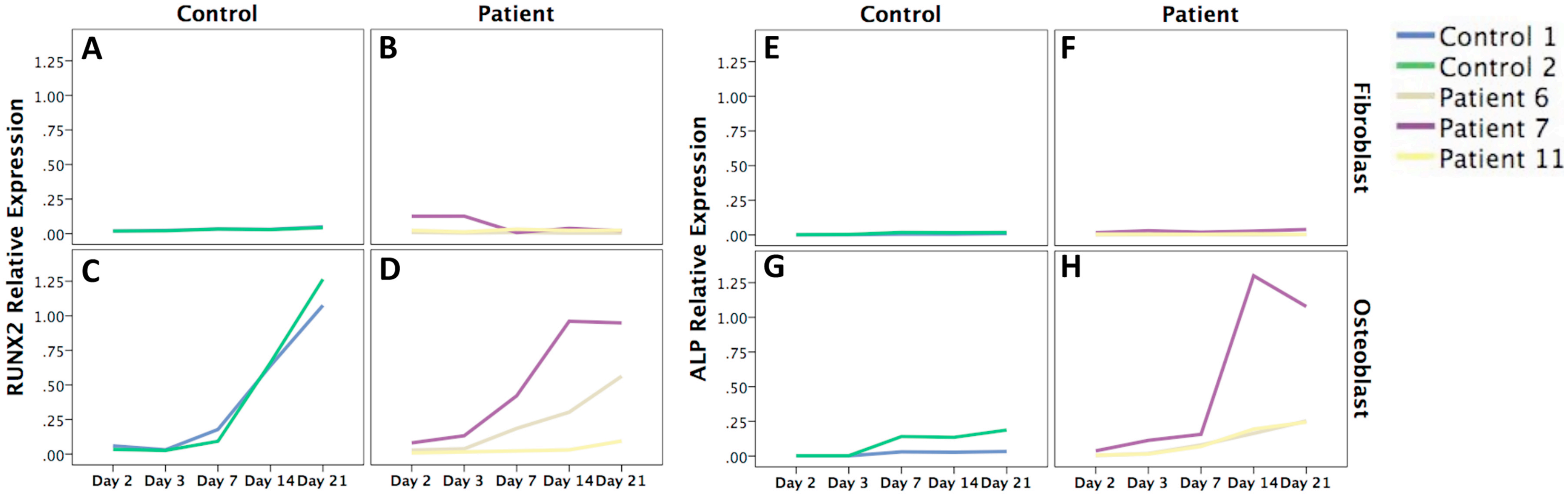


Relative expression of osteogenic markers (*RUNX2* and *ALP*) after day 2, 3, 7, 14 and 21 of culture in osteogenic media supplemented with platelet lysate shows all controls and patients fibroblasts cells are transdifferentiated to osteoblasts. (A-D) Expression of *RUNX2* relative to *YWHAZ* (housekeeping gene) of cells cultured in fibroblast media from controls (A) and patients (B) and transdifferentiated osteoblasts from controls (C) and patients (D). (E-G) Expression of *ALP* relative to *YWHAZ* of cells cultured in fibroblast media from controls (E) and patients (F) and transdifferentiated osteoblasts from controls (F) and patients (G).

**Supplemental Figure 2.**

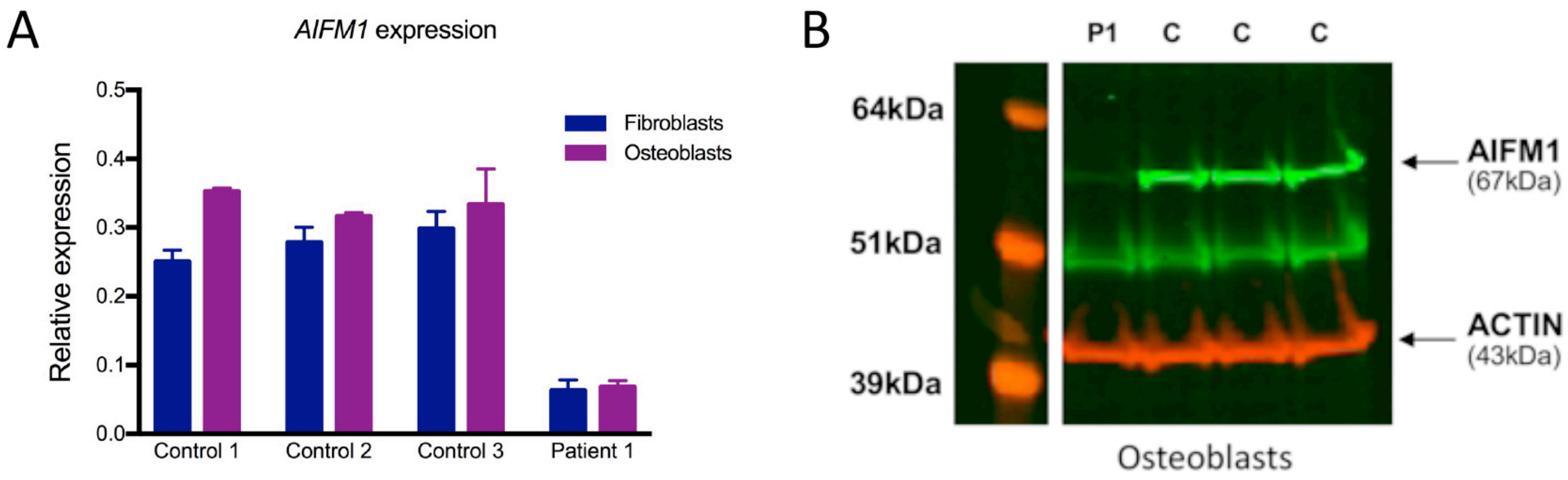


In (A), *AIFM1* mRNA expression normalized to housekeeping gene *YWAHZ* is depicted, showing clearly reduced *AIFM1* mRNA in fibroblasts and osteoblasts of patient 1 (carrying the synonymous c.720C>T mutation). (B) Western blot of AIFM1 and Actin in transdifferentiated osteoblasts (day 21) using cells of patient 1. These findings are similar to the results found in patients 7, 6 and 11. kDa: kiloDalton

**Supplemental figure 3.**

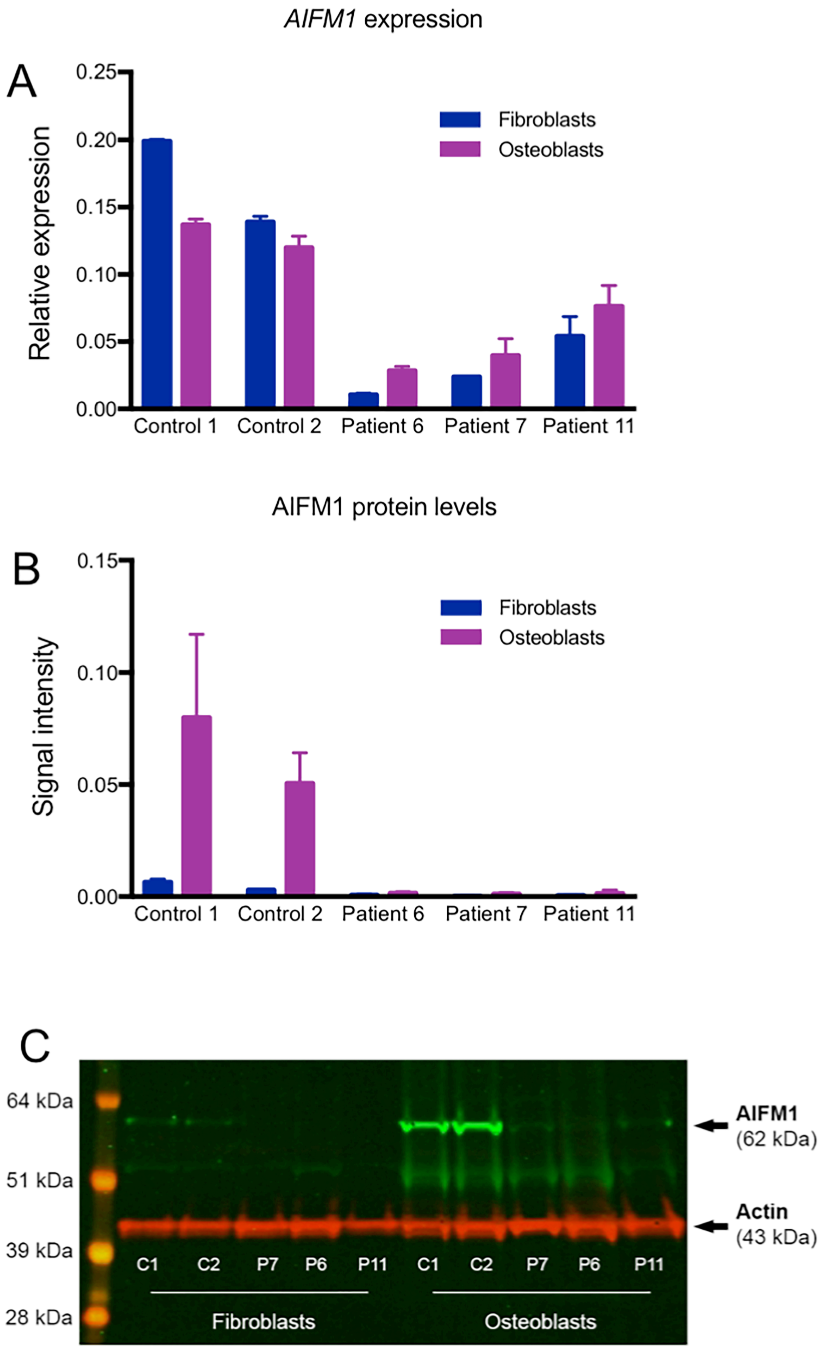


Expression of AIFM1 mRNA (A) and protein (B, C) in fibroblasts and osteoblasts of patients 6, 7 and 11. Note the almost complete absence of AIFM1 protein in fibroblasts compared to osteoblasts (B, C).

## References


1. Miyake, N., Tsukaguchi, H., Koshimizu, E., Shono, A., Matsunaga, S., Shiina, M., Mimura, Y., Imamura, S., Hirose, T., Okudela, K., et al. (2015). Biallelic Mutations in Nuclear Pore Complex Subunit NUP107 Cause Early-Childhood-Onset Steroid-Resistant Nephrotic Syndrome. Am J Hum Genet 97, 555-566.
2. Naaijkens, B.A., Niessen, H.W., Prins, H.J., Krijnen, P.A., Kokhuis, T.J., de Jong, N., van Hinsbergh, V.W., Kamp, O., Helder, M.N., Musters, R.J., et al. (2012). Human platelet lysate as a fetal bovine serum substitute improves human adipose-derived stromal cell culture for future cardiac repair applications. Cell Tissue Res 348, 119-130.
3. Micha, D., Voermans, E., Eekhoff, M.E., van Essen, H.W., Zandieh-Doulabi, B., Netelenbos, C., Rustemeyer, T., Sistermans, E.A., Pals, G., and Bravenboer, N. (2016). Inhibition of TGFbeta signaling decreases osteogenic differentiation of fibrodysplasia ossificans progressiva fibroblasts in a novel in vitro model of the disease. Bone 84, 169-180.
